# Mitochondrial DNA – novel mechanisms of kidney damage and potential biomarker

**DOI:** 10.1097/MNH.0000000000000922

**Published:** 2023-09-01

**Authors:** Afshan N. Malik

**Affiliations:** King's College London, Diabetes and Obesity, School of Cardiovascular Medicine and Metabolic Sciences, Guy's Campus, London, UK

**Keywords:** acute renal injury, biomarker, chronic kidney disease, mitochondrial DNA

## Abstract

**Purpose of review:**

MtDNA copy number (CN), a putative noninvasive biomarker of mitochondrial dysfunction, is associated with renal disease. The purpose of this review is to describe studies which measured human blood mtDNA-CN in the context of chronic kidney disease (CKD), and to evaluate its potential as a clinical biomarker of kidney disease.

**Recent findings:**

Following on from small scale cross-sectional studies implicating mtDNA-CN changes in diabetic kidney disease, recent large scale population studies provide compelling evidence of the association of mtDNA-CN and risk of renal disease in the general population and poor outcomes in CKD patients.

**Summary:**

The kidney has high bioenergetic needs, renal cells are rich in mitochondrial content containing 100s to 1000s of mtDNA molecular per cell. MtDNA has emerged as both a potential mediator, and a putative biomarker of renal disease. Damage to mtDNA can result in bioenergetic deficit, and reduced MtDNA levels in the blood have been shown to correlate with CKD. Furthermore, leakage of mtDNA outside of mitochondria into the cytosol/periphery can directly cause inflammation and is implicated in acute kidney injury (AKI). Recent large-scale population studies show the association of mtDNA-CN and renal disease and provide a strong basis for the future evaluation of circulating DNA-CN in longitudinal studies to determine its utility as a clinical biomarker for monitoring renal function.

## INTRODUCTION

Chronic kidney disease (CKD) is a serious world-wide health problem, which can progress to end-stage kidney disease (ESKD) and increases the risk of poor health outcomes, including cardiovascular disease and mortality [1]. The global rise in diabetes and obesity, major risk factors for CKD, makes the search for diagnosis and monitoring of CKD an extremely urgent health issue [2]. CKD is characterized by long periods of clinical silence, often decades, when patients are asymptomatic and there are no markers available to detect the underlying pathology. The current methods of diagnosis are only possible after significant renal damage, detected either as albuminuria, the leakage or protein into the urine, or as decline in the glomerular filtration rate (GFR), the latter most often being estimated [3]. Albuminuria is measured as the albumin/creatinine ratio (ACR), and classified as three progressive categories A1, A2, and A3. However, ACR can miss a significant proportion of patients at risk of renal failure who remain normoalbuminuric. GFR is determined as estimated glomerular filtration rate (eGFR) of <60 ml/min/1.73 m^2^, levels, defined as kidney failure when it reaches <15 ml/min/1.73 m^2^. The loss of eGFR over time has some predictive value but is inconsistent, and the prediction of eGFR loss based on clinical and demographic risk factors is poor. The refinement of an individual's CKD progression risk has been partially improved by The Kidney Disease Improving Global Outcomes (KDIGO) classification, which combine eGFR with ACR, and categorizes the risk of CKD progression into four groups (low, moderate, high, or very high risks) [4,5]. Despite these improvements, there remains a strong clinical need for biomarkers to identify patients at risk before organ damage and a lack of clinically useful biomarkers that could assist in monitoring renal disease progression [6,7].

A landmark hypothesis proposed by Douglas Wallace in the 1990s suggested that mitochondrial dysfunction and metabolic deficit resulting from damage to mitochondrial DNA (mtDNA) underlie many common aging and degenerative diseases [8,9]. However, despite increasing evidence of mitochondrial dysfunction being involved in many common diseases such as diabetes, cardiovascular disease, and more recently kidney disease, measuring mitochondrial function in humans has remained elusive due to the lack of suitable biomarkers. In 2011, we proposed the use of circulating mtDNA-CN as a minimally invasive biomarker of mitochondrial dysfunction after finding in an earlier study in 2009 that it was altered in patients with diabetic nephropathy [10–12]. Since then, the correlation between changing mtDNA levels in the blood and renal disease has been supported by several studies. In this review, I will discuss the potential underlying mechanisms involved in the changes in mtDNA-CN in CKD and describe the emerging evidence from recent studies of human populations using blood samples which show the potential of circulating mtDNA-CN as a risk biomarker for kidney disease, highlighting the gaps in the current literature. 

**Box 1:**
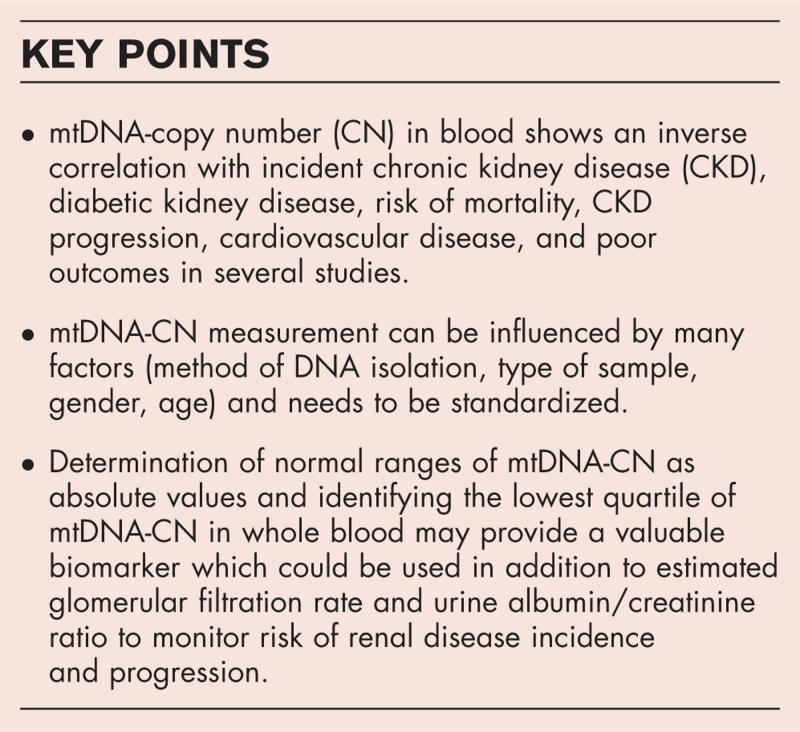
no caption available

## MITOCHONDRIA, THE ENERGY PRODUCING ORGANELLES

Mitochondria are eukaryotic organelles present in the cytosol of all nucleated eukaryotic cells with multiple key functions of fundamental importance to cellular health. The kidneys are particularly rich in mitochondrial content and therefore sensitive to the impact of mitochondrial dysfunction.

### Mitochondrial function and structure

Mitochondria are the major cellular site for the generation of energy in the form of ATP, and regulate multiple other cellular functions including apoptosis, calcium homeostasis, cellular differentiation, synthesis of key macromolecules, and growth [13]. Mitochondria are both the major site of most of the cells’ reactive oxygen species (ROS), and packed full of the cells’ endogenous antioxidants. Consequently, they play a key role in maintaining the redox balance of the cell and hence regulate multiple signalling pathways. The individual mitochondrion has a discrete double membrane structure, comprising of an outer membrane (OMM), which governs selective transport in and out of mitochondria, and an inner membrane (IMM) which is folded to produce cristae, providing a larger surface area due to the folds. Large protein complexes collectively known as the electron transport chain are embedded within the IMM, which surrounds the inner matrix of the organelle. The electron transport chain is comprised of four highly conserved protein complexes which couple redox reactions, creating a chemical gradient which leads to the conversion of oxygen to carbon dioxide and the generation of ATP via a process known as oxidative phosphorylation. Under normal physiological conditions, instead of being discrete individual organelles, most of the cells’ mitochondria exist as an interconnected network, a result of mitochondrial fusion, allowing individual mitochondria to share internal components [13].

### Mitochondrial DNA

Mitochondria are the only organelle outside of the nucleus which contain their own DNA genome known as mitochondrial DNA (mtDNA), found complexed with TFAM in the IMM. The mitochondrial genome, a 16.5 kb DS DNA molecule, was the first human genome sequenced in 1981 and shown to contain 13 protein coding genes, 22 tRNAs and 2 rRNAs [14]. In addition, emerging data suggests the presence of multiple other alternative mtDNA transcripts of functional significance, for example humanin [15]. The 13 protein coding genes in mtDNA are key subunits of the electron transport chain and therefore essential for the oxidative phosphorylation process. The replication of the 16.5 kb circular mtDNA genome is independent of the nuclear genome and resembles bacterial DNA in terms of its methylation status. Each mitochondrion can house 2–10 copies of the 5-μm 16.5 kb circular mtDNA genome. This mtDNA is replicated, transcribed, and translated within mitochondria. The translation of the 13 protein coding genes in mtDNA uses a slightly different genetic code to the cells’ nuclear universal genetic code (reviewed in [12]).

### Mitochondrial life cycle

Most cells are constantly making new mitochondria and removing damaged mitochondria, a process known as the mitochondrial life cycle. Damaged mitochondria and their mtDNA are specifically degraded and removed, and mitochondrial biogenesis, formation of new mitochondria, requires the replication of existing mtDNA as a template and then distributed to new organelles through fission and fusion. mtDNA replication requires specific transcription factors with the main transcription factor being TFAM, a protein which forms a complex with mtDNA in the form of a nucleoid in the IMM. Both the mitochondrial and nuclear genomes are intricately involved in mitochondrial biogenesis and the mitochondrial life cycle, since most of the proteins needed to make functional mitochondria are transcribed in the nuclear genome. Nuclear genome encoded mitochondrial proteins are translated in cytosolic ribosomes, and transported to mitochondria where they are either used to drive functional processes or used as structural components of the mitochondrial organelle (reviewed in [12]).

## ASSESSING MITOCHONDRIAL HEALTH AND MITOCHONDRIAL DYSFUNCTION

Cellular health is intricately linked with mitochondrial health and mitochondrial dysfunction can therefore affect multiple fundamental cellular functions. It is not surprising that in recent years mitochondrial dysfunction has emerged as of major importance in many human diseases including renal disease.

### Circulating mitochondrial DNA copy number as a biomarker

**FIGURE 1:**
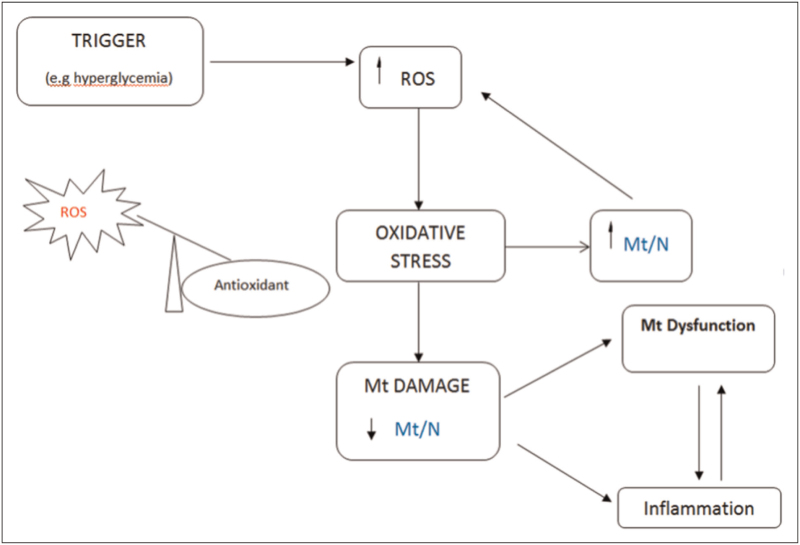
Proposed mechanism underlying changes in mitochondrial DNA-copy numbers (CN). A schematic showing potential mechanisms which may lead to decreased mtDNA-CN. A trigger (e.g. hyperglycemia) leads to increased intracellular reactive oxygen species (ROS). Under normal conditions, the cell's endogenous antioxidant response recovers the redox balance by scavenging the ROS. However, under conditions of chronic ROS, the cell's antioxidant response is overwhelmed resulting in oxidative stress. Increased intracellular ROS may initially lead to increased mitochondrial biogenesis, an adaptive response, detected by measuring Mt/N (mitochondrial to nuclear genome ratio—also described as cellular mtDNA-CN). Over time the oxidative stress would cause damage to mitochondrial membranes, proteins and DNA leading to mitochondrial dysfunction, reduced Mt/N, and cause an energy deficit. Accumulation of damaged MtDNA can also cause an inflammatory response as MtDNA is un-methylated and resembles bacterial DNA. Original figure—adapted from Malik and Czajka [12].

We originally proposed the use of circulating mtDNA-CN, measured as the mitochondrial genome to nuclear genome ratio (Mt/N), as a noninvasive biomarker of mitochondrial dysfunction [10] and suggested that it may be useful in diseases where mitochondrial dysfunction is implicated if the methodological issues which led to some earlier erroneous reports were avoided [10,12]. The potential underlying mechanisms implicated in mitochondrial dysfunction were proposed as the Mt/N hypothesis [12] (Fig. 1), which provided a mechanistic framework for the changes observed in MtDNA-CN in disease (discussed below). In the last 2 decades, thousands of studies have been published using clinical samples which measure variations in mtDNA-CN in numerous human diseases, discussion of these are beyond the scope of the current review which is focused primarily on CKD. However, it is important to mention that when measuring mtDNA-CN from blood, there are several important considerations. Studies need to clarify whether cellular or cell free mtDNA is being measured using qPCR [16^▪▪^,^17^], or if mtDNA is being estimated [18] as well as the influence of blood cell composition [19^▪^], since all of these and other methodological factors can influence the resultant data.

### Variations in mitochondrial DNA content

Depending on the cells’ bio-energetic requirements, the numbers of mitochondria can vary between cells, from hundreds to thousands, however the mechanisms by which cells with higher bioenergetic needs have higher numbers of mitochondria are not understood [20]. As MtDNA content largely correlates with mitochondrial number, it can be used to assess mitochondrial content of different cell and sample types. Cellular mtDNA levels can also change in response to physiological stimuli and conditions of stress [12]. The exact mtDNA-CN ranges in different cells and tissues are not clearly established but some studies have reported the ranges. Blood cells may contain 50–100 mtDNA copies per cell [17,21] whereas regions of the human brain have thousands of copies of mtDNA per cell [22]. In a study looking at mouse tissues, the heart, kidney, and brain had the highest mtDNA-CN, correlating with the metabolic requirements of these organs and resembling proteomic studies showing similar abundance levels [23].

### Circulating mitochondrial DNA copy number in whole blood and blood fractions

**FIGURE 2:**
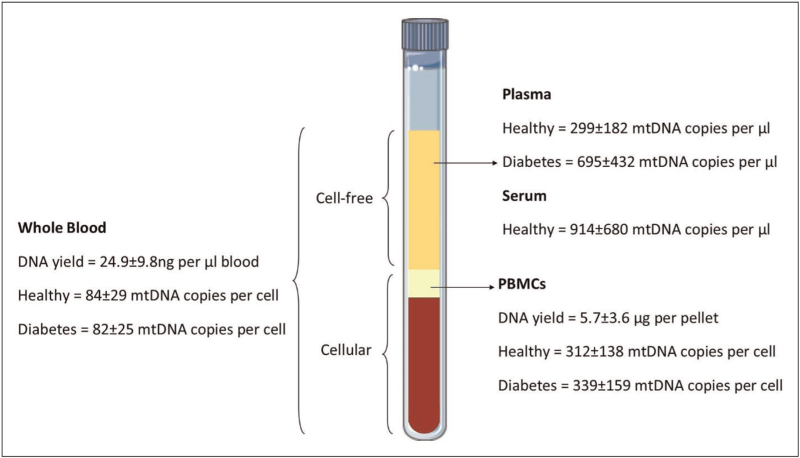
Normal and abnormal ranges of mitochondrial DNA in human blood. Circulating cellular and cell-free mtDNA in human blood. Schematic summarizing data for total genomic DNA yields and mitochondrial DNA content for whole peripheral blood and its cellular (PBMCs) and cell-free (plasma and serum). Previously published figure from Rosa and Malik [16^▪▪^].

To consider whether a blood test based on mtDNA-CN may be developed which can help in the prediction of risk of CKD occurrence or progression, I have restricted the studies I reviewed to those using human blood samples. MtDNA-CN can be measured from whole blood, buffy coat, purified peripheral blood mononuclear cells (PBMCS), or from cell free fractions of blood (plasma or serum). We have previously published the values of mtDNA-CN found in these different fractions in healthy controls and diabetes patients [16^▪▪^,^17^] as shown in Fig. 2. mtDNA-CN can be expressed as cellular mtDNA, normalized to the nuclear genome, previously described by us as the Mt/N ratio, or as cell free mtDNA, normalized to the volume of serum or plasma, described as mtDNA copies/ul. [16^▪▪^,^17^,^21^]. This approach requires the determination of absolute copy numbers in human blood normalized either to cell numbers or to volume of blood sample and can allow comparisons between different studies. Additionally, large scale population studies have determined estimated mtDNA-CN using probe densities from microarrays or other strategies to obtain the information from existing datasets, in these studies the values given to mtDNA-CN are largely arbitrary and can be derived from multiple sample types including blood and tissue samples and are normalized as relative amount of change in groups for comparisons [18].

## THE KIDNEYS AND MITOCHONDRIAL DYSFUNCTION

Kidneys are organs requiring large amounts of energy in form of ATP due to the re-absorption processes and although their mass accounts for less than 1% of total body mass, they use almost 10% of the body's oxygen which is utilized in cellular respiration via OXPHOS, and therefore they are rich in mitochondrial content and MtDNA [24,25].

### Early studies showing mitochondrial involvement in renal disease

There were studies pertaining to mitochondrial involvement in renal disease many years before circulating mtDNA-CN started to be used as a biomarker. For example, mitochondrial circulating antibodies were reported in patients with end stage renal disease and nephrotoxicity in the 1980s [26,27], many studies reported the association of mtDNA deletions in primary genetic mitochondrial diseases [28] and kidney involvement in mitochondrial disorders [29]. Taking it beyond primary mitochondrial genetic disease, Douglas Wallace proposed that damage to mtDNA, such as mutations and/or deletions, and effects of mtDNA haplotypes may be involved in human degenerative diseases and aging [30]. Suzuki *et al.*[31] suggested that oxidative damage to muscle mtDNA was involved in diabetic complications, subsequently several studies focused on mtDNA damage and mtDNA mutations most often from tissue rather than blood. Studies measuring mtDNA-CN appeared later and followed on from many studies showing links to MtDNA mutations, haplotypes, and mitochondrial dysfunction in some renal and many other disorders. A 4977 bp mtDNA deletion was reported in blood samples from CKD patients in 2008 [32]. A report measuring mtDNA-CN in renal tissue showed reduced mtDNA-CN and energy metabolism [33].

### The potential mechanisms underlying mtDNA mediated renal dysfunction

mtDNA is present in all nucleated cells and present in high numbers in cells with high bioenergetic needs such as renal cells. Measurement of mtDNA-CN in blood cells can be viewed as a surrogate for systemic mtDNA levels including in the kidney [34]. The potential mechanisms that lead to renal dysfunction can be extrapolated from the hypothesis shown in Fig. 1. We used human primary renal glomerular mesangial cells (HMCs) as well as patient samples to detect the changes predicted by the hypothesis [35^▪▪^]. We showed that when HMCs are grown in diabetic conditions, the high glucose leads to increased ROS and at the same time we observed more than 200% increase in cellular MtDNA, however this MtDNA was damaged, and moreover continued growth in high glucose led to reduced mtDNA-CN, and damaged cellular respiration in parallel with increased ROS [35^▪▪^]. We also found evidence of similar changes in blood samples taken from diabetes patients with and without diabetic kidney disease [35^▪▪^].

## CIRCULATING mtDNA-CN CHANGES IN CHRONIC KIDNEY DISEASE

In this section we will review human studies which link aspects of renal function with changes in circulating mtDNA-CN (Table 1).

**Table 1:** Studies suggesting circulating mtDNA-CN as a biomarker of kidney disease/renal function in the context of renal function and chronic kidney disease

Method of mtDNA-CN determination	Patient population (sample type/numbers)	Key points	Reference
Relative quantification delta CT method	T2D South Asian (cross-sectional study, whole blood *n* = 60)	MtDNA-CN higher in patients with T2DN relative to T2D no renal disease (no healthy controls, primers that co-amplify NUMTS)	Malik *et al.*[11]
Relative quantification deltaCT method	Community based general population study (leukocytes, *n* = 694))	The prevalence of microalbuminuria decreased progressively from lower to upper quartiles of mtDNA-CN	Lee *et al.*[37^▪^]
Absolute quantification using qPCR	Diabetes clinic London: T1D and T2D ± nephropathy (cross sectional study, whole blood, *n* = 169)	MtDNA-CN lower in patients with DKD, alongside increased mtDNA damage, reduced metabolic flexibility and altered mitochondrial RNAs.	Czajka *et al.*[35^▪▪^]
Affymetrix arrays; estimated mtDNA-CN	Atherosclerosis Risk in Communities Study Longitudinal study over 19.6 years, *n* = 9058	Higher e-mtDNA-CN associated with lower risk of incident CKD (highest versus lowest quartile: hazard ratio 0.65; 95% confidence interval, 0.56 to 0.75; *P* = 0.001)	Tin *et al.*[38^▪▪^]
Absolute quantification using plasmid based qPCR	German Chronic Kidney Disease (GCKD) study, *n* = 4812, cross sectional and longitudinal study	Lowest quartile of mtDNA-CN showed highest risk of mortality and infections, 4 years follow up	Fazzini *et al.*[39^▪▪^]
Illumina HumanOmni 1-Quad Array. Estimated mtDNA-CN	Chronic Renal Insufficiency Cohort study (CRIC) Longitudinal study *N* = 2943	Lowest tertile of e-mtDNA-CN showed the highest risk of progression, 6.5 years follow up	He *et al.*[40^▪▪^]
Relative mtDNA-CN Taqman probes (delta Ct method)	Type 2 diabetes patients with progressive stages of renal disease *N* = 180	Linked serum (and urinary) mtDNA to inflammatory status in patients with diabetic kidney disease	Petrica *et al.*[36]

Note: excludes studies reporting mtDNA-CN changes in AKI, IGA nephropathy and hemodialysis patients. Excludes studies reporting mtDNA mutations or measuring mtDNA in renal/other tissues. AKI, acute kidney injury; CN, copy number.

### Cross sectional studies with diabetic kidney disease

An early and possibly the first report of circulating mtDNA in CKD was from our lab in 2009 where we measured, in whole blood, mtDNA-CN using the then established method of real time qPCR and primers which subsequently turned out to be not specific to the mitochondrial genome but which could amplify nuclear mitochondrial insertion sequences (NUMTs) [10,11] In this small cross-sectional study we found that relative mtDNA levels were increased in T2D patients with nephropathy compared to those with no kidney disease. Subsequently, using absolute quantification, we reported that reduced circulating mtDNA-CN correlated with diabetic kidney disease [35^▪▪^]. Furthermore, PBMCs from patients with diabetic kidney disease also showed reduced bioenergetic flexibility and increased mtDNA damage [35^▪▪^], confirming the presence of mitochondrial dysfunction. A recent study that showed the potential functional consequence of blood mtDNA-CN changes in patients with diabetic kidney disease was by Petrica *et al.*[36], they showed that serum (and urinary) mtDNA-CN changes correlated with an inflammatory signature in their patients with increasing renal dysfunction.

### Association with albuminuria in a community-based study

A cross-sectional community-based study reported peripheral blood mtDNA-CN in 694 adults with normal renal function using real time qPCR [37^▪^]. Their population had a prevalence of microalbuminuria (ACR > 30 mg/g) of 4.5% which decreased progressively from the lower to the upper quartiles of mtDNA-CN. Surprisingly, their mtDNA-CN ranges were much higher than those reported in earlier studies using whole blood [16^▪▪^,^17^,^21^]. The authors mention in the methods section that DNA was from leukocytes was used to measure mtDNA-CN, therefore it is possible that fractionation was carried out to isolated PBMCs [37^▪^]. Although this information is not provided in the paper, it might explain the relatively high mtDNA-CN absolute copy numbers they obtained compared to in whole blood, since their median copy number range in their population was reported to be 467 (228–928) per cell. The study by Lee *et al.* is important in showing a trend for lower mtDNA-CN associated with higher microalbuminuria and hence and increased risk of CKD progression.

### Mitochondrial DNA copy number and risk of incident chronic kidney disease

The next key report was a large cohort study showing an association between mtDNA-CN and incident CKD in the Atherosclerosis Risk in Communities Study [38^▪▪^]. The authors estimated mtDNA-CN in peripheral blood using 25 high-quality mitochondrial single nucleotide polymorphisms from the Affymetrix 6.0 array in 9058 participants, making this one of the largest studies of mtDNA-CN at this scale which reported a link between incident CKD and mtDNA-CN. The blood samples used were described as buffy coat from which genomic DNA was isolated and hybridized to Affymetrix 6.0 microarrays. Twenty five high quality mtDNA SNPs were identified which were said to not cross hybridize to the NUMTs in the nuclear genome. This allowed the authors to use probe densities which were standardized using a linear model which corrected for DNA quality, quantity, batch effects and GC content to estimate relative mtDNA-CN. 1490 participants developed CKD over a median follow-up of 19.6 years. There was no association with mtDNA-CN and eGFR at baseline, but higher mtDNA-CN associated with lower risk of incident CKD (highest versus lowest quartile: hazard ratio 0.65; 95% confidence interval, 0.56–0.75; *P* = 0.001) after adjusting for age, sex, and race. After adjusting for additional risk factors of CKD, including prevalent diabetes, hypertension, C-reactive protein level, and white blood cell count, this association remained significant (highest versus lowest quartile: hazard ratio 0.75; 95% confidence interval, 0.64–0.87; *P* = 0.001). The authors suggested that future research should focus on modifiable risk factors that improve mtDNA-CN. Although this study shows an association, the authors did not provide any information on the relationship between the estimated mtDNA-CN using mtDNA SNPS and the corresponding absolute mtDNA-CN [38^▪▪^]. The data generated are arbitrary numbers based on probe densities, the potential use of mtDNA-CN as a biomarker requires the determination of the normal and abnormal values of mtDNA-CN in the population under study to identify potential risk ranges. Nevertheless, the scale of this study provides excellent robust support for both a potential mechanistic role of mtDNA-CN changes in the risk of incident CKD, as well as highlighting the promise of mtDNA-CN as a risk marker.

### Mitochondrial DNA copy number in a large chronic kidney disease cohort predicts poor outcome

An excellent study which has gone some way to resolving the question of normal and abnormal ranges came from a well established large cohort of 4812 patients from the German Chronic Kidney Disease (GCKD) study, an ongoing prospective multicentre observational study which enrolled patients under the regular care of nephrologists for whom good clinical information especially in terms of renal function was available [39^▪▪^]. Unlike other large cohort studies which estimated mtDNA-CN, Fazzini *et al.* used a highly accurate plasmid based real time PCR quantification assay allowing them to determine absolute values of mtDNA-CN, which they reported as between 21.29 and 379.5 per cell for CKD patients at stages G3 or G1-2,A3. They showed a 1.8-fold increase in mortality in patients with the lowest quartile of mtDNA-CN and this effect was independent of renal function and cardiovascular disease. They also showed an increased risk of cardiovascular disease as well as nearly double the risk of hospitalization and infection in the lowest quartile of mtDNA-CN. Their data strongly supports the future use of mtDNA-CN as a clinically useful tool for assessing risk by identifying patients in the lowest quartile and determining whether this can be modified.

### Mitochondrial DNA copy number in a chronic kidney disease population and risk of disease progression

Another study looking at a large CKD cohort was by He *et al.*[40^▪▪^] who assessed the prospective association of mtDNA-CN with CKD progression. They used 2943 participants from the Chronic Renal Insufficiency Cohort study (CRIC) and estimated mtDNA-CN from probe intensities of mitochondrial single nucleotide polymorphisms. They found that the lowest tertile of estimated mtDNA-CN showed the highest risk of progression, again providing strong support for the use of mtDNA-CN as a risk biomarker. However, they did not determine absolute mtDNA-CN, and as they estimated mtDNA-CN in the serum of the patients, this would represent cell free mtDNA, a different blood fraction from the other studies described in this section.

### Mitochondrial DNA copy number changes and other common renal diseases

As well as the above studies on circulating mtDNA-CN largely related to CKD or renal function, many studies have reported changes in both circulating and urinary mtDNA-CN associated with other renal disorders but which are beyond the scope of the current article. Interestingly, in a recent study (Liu *et al.*, 2023), reduced peripheral blood mtDNA-CN was linked to low GFR (eGFR< 60 mL/min/1.73 m^2^) in patients with biopsy proven immunoglobulin A nephropathy [41]. Urinary mtDNA has been linked to kidney dysfunction, and specifically acute kidney injury in several studies but to date there are few large-scale population studies. There are likely similar mechanisms underlying systemic mitochondrial dysfunction which leads to changes in mtDNA-CN in other disorders of the kidney and it will be interesting to see whether mtDNA-CN could potentially be a biomarker of renal impairment in addition to the existing clinical measures of eGFR and urine ACR.

## CONCLUSION

In this article, the focus was on evaluating studies which have measured circulating mtDNA-CN in the context of CKD, as well as population studies which found a link with aspects of renal function and changes in mtDNA-CN. The evidence that reduced circulating mtDNA-CN indicates risk of renal function decline as well as poor outcome is increasing, and recent large-scale studies have shown strong negative association between mtDNA-CN and health outcomes in the CKD population. However, apart from a few studies which have measured absolute levels, we still do not have established normal and ‘at risk’ levels of mtDNA-CN. This issue is confounded by studies using different sample types. There is a lack of studies measuring the change in mtDNA-CN over time, this is important to determine whether mtDNA-CN has prognostic value in predicting future loss of renal function. There is a strong argument for undertaking such studies as routine monitoring of mtDNA-CN in the CKD population could provide an additional marker to the existing KDIGO, ACR and eGFR based methods. As a DNA based marker which can be measured accurately using very small volumes of whole blood, as little as 10ul, using qPCR to determine values, the use of mtDNA-CN could provide a fast, accessible, and economical biomarker for clinical use.

## Acknowledgements

*None.*

### Financial support and sponsorship

*None.*

### Conflicts of interest

*There are no conflicts of interest.*

## REFERENCES AND RECOMMENDED READING

Papers of particular interest, published within the annual period of review, have been highlighted as:

▪ of special interest
▪▪ of outstanding interest
